# Ocurrence of *Nyssomyia intermedia* (Diptera: Psychodidae) in intradomiciliary environments: a potential vector of american cutaneous leishmaniasis in Montezuma, 2024

**DOI:** 10.1590/S2237-96222025v34e20250198.en

**Published:** 2025-10-27

**Authors:** Bartolomeu Teixeira Lopes, Cirilo Henrique de Oliveira, Pedro Augusto Almeida de Souza, Rudá Mahayana Cordeiro-de-Barros, Thaynara de Jesus Teixeira, Ronaldo Medeiros dos Santos, Agna Soares da Silva Menezes, Filipe Vieira Santos de Abreu

**Affiliations:** 1 Secretaria de Saúde de Minas Gerais, Superintendência Regional de Saúde de Montes Claros, Montes Claros, MG, Brazil; 2 Instituto Federal do Norte de Minas Gerais, Laboratório de Comportamento de Insetos, Salinas, MG, Brazil; 3 Instituto Federal do Norte de Minas Gerais, Departamento de Engenharia Florestal, Salinas, MG, Brazil; 4 Secretaria Municipal de Saúde de Salinas, Centro Colaborador de Entomologia/Lacoi/IFNMG, Salinas, MG, Brazil

**Keywords:** Psychodidae, Neglected Diseases, Entomology, Public Health Surveillance, Leishmaniasis, Cutaneous, Psychodidae, Enfermedades Desatendidas, Entomología, Vigilancia en Salud Pública, Leishmaniasis Cutánea

## Abstract

**Objective:**

To investigate the Phlebotominae fauna in the rural area of Montezuma, Minas Gerais, following the confirmation of a human case of American cutaneous leishmaniasis, in collaboration with the Regional Health Superintendent’s Office of Montes Claros.

**Methods:**

The captures were carried out with CDC-type light traps (developed by the Centers for Disease Control and Prevention, CDC), which were placed in the intradomiciliary area (bedroom 1 and bedroom 2) and the peridomestic area (chicken coop) during two consecutive nights.

**Results:**

In total, 30 phlebotomine sand flies were captured and identified, with 29 (96.67%) belonging to the species *Nyssomyia intermedia* and 1 (3.33%) belonging to the species *Psathyromyia pascalei*. Surprisingly, all specimens of *Nyssomyia intermedia* were captured in the intradomiciliary area, specifically in the patient’s bedroom, with confirmed American cutaneous leishmaniasis infection.

**Conclusion:**

Due to the large number of specimens captured inside the residence, *Nyssomyia intermedia* stands out as a potential intradomiciliary vector of American cutaneous leishmaniasis. In addition, we reinforce the importance of studies such as this one, conducted in collaboration between the Brazilian National Health System (*Sistema Único de Saúde*, SUS) and the Collaborating Centers of Minas Gerais, to strengthen surveillance actions, promote preventive measures, and expand knowledge about the ecoepidemiology of the disease in areas still little studied.

## Resumen

**Objetivo:**

Investigar la fauna de flebótomos en la zona rural de Montezuma, Minas Gerais, tras la confirmación de un caso humano de leishmaniasis tegumentaria americana, en una acción conjunta con la Superintendencia Regional de Salud de Montes Claros.

**Métodos:**

Las capturas se realizaron mediante trampas luminosas tipo CDC (desarrolladas por los Centers for Disease Control and Prevention), colocadas en el interior de la vivienda (habitación 1 y habitación 2) y en el peridomicilio (gallinero) durante dos noches consecutivas.

**Resultados:**

En total se capturaron e identificaron 30 flebótomos, de los cuales 29 (96,67 %) pertenecían a la especie *Nyssomyia intermedia* y 1 (3,33 %) a la especie *Psathyromyia pascalei*. Sorprendentemente, todos los ejemplares de *Nyssomyia intermedia* fueron capturados en el interior de la vivienda, específicamente en la habitación del paciente con infección confirmada por leishmaniasis tegumentaria americana.

**Conclusión:**

Debido al elevado número de especímenes capturados dentro de la vivienda, Nyssomyia intermedia se destaca como un vector potencialmente intradomiciliario de la leishmaniasis tegumentaria americana. Además, se refuerza la importancia de estudios como este, realizados en colaboración entre el Sistema Único de Salud (*Sistema Único de Saúde*, SUS) y los Centros Colaboradores de Minas Gerais, para fortalecer las acciones de vigilancia, promover medidas profilácticas y ampliar el conocimiento sobre la ecoepidemiología de la enfermedad en áreas aún poco estudiadas.

**Ethical aspects**

This research used public domain anonymized databases.:

## Introduction

Phlebotominae sand flies (Diptera: Psychodidae, Phlebotominae) are small insects, commonly known as sand flies, that play a crucial role in the transmission of pathogens to humans. They are responsible for the transmission of different species of intracellular protozoa of the genus Leishmania Ross, the causative agents of leishmaniasis (1). The genus Leishmania includes more than 20 species transmitted among mammalian hosts through the bites of female sand flies during hematophagy. In most cases, the transmission cycle is zoonotic, involving one or more non-human animal reservoirs (2). In Brazil, the *Lutzomyia longipalpis* species complex serves as the primary vector of visceral leishmaniasis, primarily caused by *Leishmania* (Leishmania) infantum. Meanwhile, *Nyssomyia intermedia* and *Nyssomyia whitmani* are the primary vectors of *Leishmania* (Viannia) braziliensis, the primary agent of American cutaneous leishmaniasis (3).

American cutaneous leishmaniasis is considered a neglected, potentially serious disease characterized by ulcerated lesions on the skin and/or mucous membranes (4). The treatment is provided by the Brazilian National Health System (SUS); however, high dropout rates are recorded due to the lengthy application period of the injections. The disease has been reported in all Brazilian states, and its epidemiological pattern has shifted, particularly in terms of transmission mode. An increase in the number of cases is observed, with recurrent peaks every five years (3). Between 2010 and 2022, 231,307 cases of American cutaneous leishmaniasis were reported in Brazil, with an annual average of 114.33 cases per 100 thousand inhabitants (5).

Minas Gerais is one of the Brazilian states with the highest rates of transmission for American cutaneous leishmaniasis. In the last decade, 16,277 cases have been recorded in the state (5). Previous studies have confirmed that American cutaneous leishmaniasis is endemic in Minas Gerais and that the northern macroregion is one of the most severely affected by the disease (6). In this region, the main vectors are *Lutzomyia longipalpis*, associated with visceral leishmaniasis (7), and *Nyssomyia intermedia*, vector of cutaneous leishmaniasis (8). Despite the high incidence of the disease and the guidelines established in leishmaniasis control protocols, there are rare cases in which entomological investigations are conducted to identify the vectors responsible for transmission. The scarcity of trained professionals and financial resources generally limits the performance of this type of study, which is fundamental for understanding the epidemiology of this disease.

Therefore, the present study describes an entomological investigation conducted after the confirmation of a case of American cutaneous leishmaniasis in a resident of the municipality of Montezuma, located in the north of Minas Gerais. The objective was to investigate the vector fauna in the affected region to identify Phlebotominae sand flies potentially involved in the transmission of the disease.

## Methods

### Study area 

**Figure 1:**
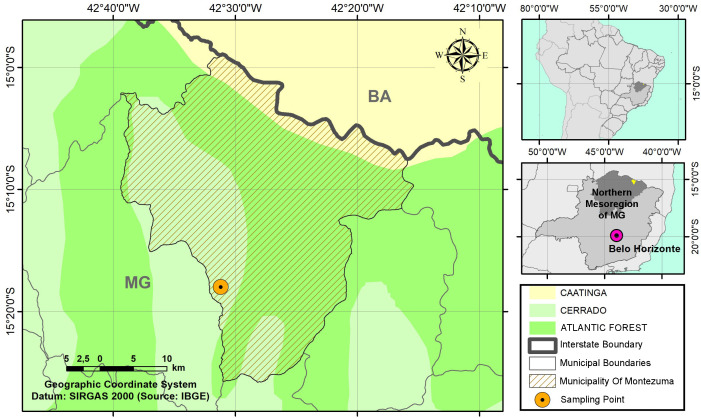
Sampling point at the intersection of three Brazilian biomes. Montezuma, 2024

The study was conducted in Montezuma, Minas Gerais, specifically in the rural community of Paz Cavalo, at Fazenda Pai Pedro (15°18’00.6”S, 42°31’21.2”W). The municipality is located in a transition area between the Cerrado, Caatinga, and Atlantic Forest biomes, in the northern macroregion of Minas Gerais (Figure 1).

Montezuma has a population of 6,888 inhabitants and a low Municipal Human Development Index (MHDI 0.587) (9). The climate of the region is classified as semi-arid (Aw, according to the Köppen classification), with an average annual precipitation of 880 mm. The climate regime is characterized by two well-defined seasons: a rainy summer, which extends from December to March, and a predominant dry season lasting throughout the remaining months of the year (10). The local vegetation is inserted in an ecotone between the Cerrado and Atlantic Forest biomes, with a predominance of plant formations and species characteristic of the Cerrado (11). 

The area was selected for entomological surveillance work due to the confirmation of a case of American cutaneous leishmaniasis in a resident, reported in May 2024. This is a rural area, with a peridomestic environment used for subsistence agriculture and livestock, including vegetable cultivation and the raising of chickens and pigs. The household where the collections were carried out was inhabited by the patient with a confirmed diagnosis of the disease and their family. The residence had no screened windows or ceiling lining and was located about 20 meters from a remnant of riparian forest on the banks of the Pardo River.

### Collection, processing, and identification of Phlebotominae sand flies

The collections were carried out at dusk and during the night between May 6 and 8, 2024. The sampling effort consisted of three CDC-type light traps, originally developed by the Centers for Disease Control and Prevention of the United States. Two of these traps were installed in the intradomiciliary area (bedroom 1 and bedroom 2), and one was placed in the peridomestic area, next to the chicken coop. The traps remained active from 18:00 to 07:00 during the two nights of collection. The captured insects were collected after each night and stored in tubes, identified by trap, containing a 70% alcohol solution. The entire collection was organized and executed by trained agents from the Regional Health Superintendent’s Office of Montes Claros.

The captured insects were sent to the Collaborating Entomology Center (*Centro Colaborador de Entomologia*, LACOI/IFNMG/Salinas). The collaborating centers were established by the State Health Department of Minas Gerais and are laboratories linked to state institutions, equipped to perform clinical and scientific analyses, providing support to the populations of their respective regions (Resolution SES/MG No. 8,441 of November 9, 2022). LACOI/IFNMG/Salinas is accredited as an “Entomology Laboratory” and is primarily responsible for conducting vector insect taxonomy, serving the 86 municipalities within the northern health macroregion of Minas Gerais (CIB/SUS-MG No. 8,441, November 9, 2022). In the laboratory, the insects were cleared in a 10% potassium hydroxide (KOH) solution for 12 hours, immersed in 100% acetic acid (CH₃COOH) for 5 seconds, and then in 10% acetic acid (CH₃COOH) for 15 minutes. Subsequently, they underwent an alcohol series with ethanol (C₂H₅OH) at concentrations of 70%, 90%, and 100%, for 10 minutes at each step. After this process, the insects were cleared in eugenol for 30 minutes, dissected, and mounted on slides with Canada balsam for subsequent microscopic examination (12). Species identification was performed using a dichotomous key based on the analysis of key morphological characters. In females, the cibarium and spermatheca were examined, while in males, the genitalia were analyzed (13). The smartphone app Lutzodex was also used, which allows species identification through real images of the specimens (14). The abbreviations of the genera followed the standardization proposed by Marcondes (15). 

After the patient’s treatment, the State Health Department of Minas Gerais implemented vector control measures, including insecticide application, and recommended the installation of window screens, as well as the use of repellents by residents, especially during twilight hours, in accordance with the guidelines of the American Cutaneous Leishmaniasis Surveillance Manual (3).

## Results

**Figure 2:**
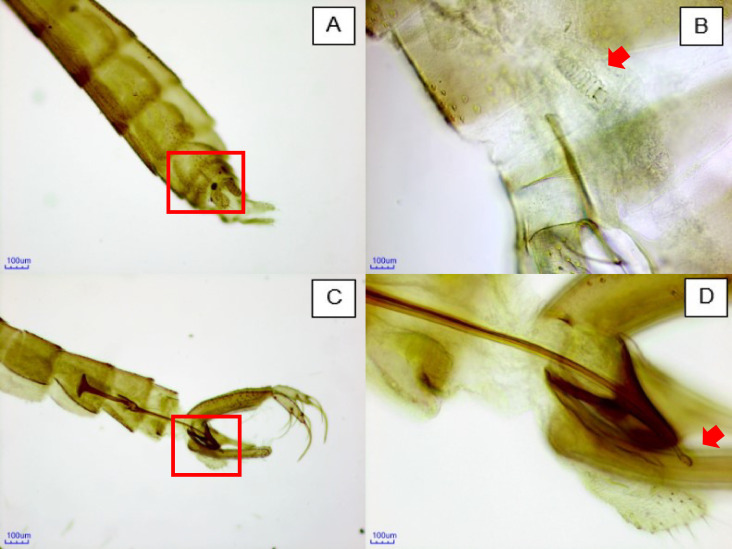
Anatomical details of the reproductive structures of *Nyssomyia intermedia*: (A) female abdomen in ventral view (200× magnification); (B) female spermatheca with anatomy consistent with *Nyssomyia intermedia* (400×); (C) terminal region of the male abdomen (200×); (D) tip of the male genital filament with anatomy consistent with *Nyssomyia intermedia* (400×). Brazil, 2024

A total of 30 Phlebotominae sand flies were captured, of which 29 (96.67%) belonged to the species *Nyssomyia intermedia* (Figure 2, A-D) and 1 (3.33%) to the species *Psathyromyia pascalei*. Surprisingly, all specimens of *Nyssomyia intermedia* were captured in the intradomiciliary environment, with most of them collected in bedroom 1, where 27 individuals (90.00%) were identified (Table 1). It is also noteworthy that the high proportion of females captured in the intradomiciliary environment, totaling 24 individuals (83.00%).

**Table 1:** Sample of phlebotomine sand fly species by number, sex, and collection site in the Paz Cavalo Community. Montezuma, 2024

Species	Collection site							
	Bedroom 1		Bedroom 2		Chicken coop			
	Female	Male	Female	Male	Female	Male	Total	%
*Nyssomyia intermedia* (Lutz & Neiva, 1912)	22	5	2	0	0	0	29	96.67
*Psathyromyia pascalei* (Coutinho & Barreto, 1940)	0	0	0	0	1	0	1	3.33
Total	22	5	2	0	1	0	30	100.0

The material was received by the Collaborating Center on 16/5/2024, and the taxonomic reports were issued on 18 and 19/5/2024 (within 72 hours after receipt). This led to a vector control response in the affected area, including environmental and health education activities coordinated by local health agents and endemic disease control workers in Montezuma. Since then, no other human cases have been reported in the region.

## Discussion

The geographical distribution of leishmaniases in Brazil reveals that its epidemiology is influenced by environmental, climatic, and socioeconomic aspects (16). American cutaneous leishmaniasis, a disease typically associated with rural environments, has changed its epidemiology driven by socio-environmental factors, such as deforestation and the migration of human populations from endemic rural areas (3). The emergence of these new risk factors has facilitated human-vector contact and generated novel epidemiological scenarios, thereby increasing the incidence of these diseases (1). 

*Nyssomyia intermedia* has a wide geographical distribution in Brazil, occurring from Piauí to Rio Grande do Sul, and is also found in parts of Argentina and Bolivia. It is predominantly reported in warm and humid regions (17). In addition, this species has already been found naturally infected with *Leishmania braziliensis*, being considered one of the main vectors of American cutaneous leishmaniasis (8). Additionally, *Nyssomyia intermedia* exhibits highly anthropophilic behavior, being generally abundant in the peridomestic areas. It was the predominant species in outbreaks of American cutaneous leishmaniasis in cities in Minas Gerais, such as Araçuaí, Lassance, and Curvelo (18). 

It is essential to note that the study area’s landscape, characterized by its proximity to remnants of natural vegetation, favors both the occurrence of *Nyssomyia intermedia* and their contact with wild reservoirs of Leishmania, such as possums and rodents, as well as other domestic hosts, including dogs and humans (3). In recent decades, it has been demonstrated that *Nyssomyia intermedia* tends to occur at high densities in secondary forest fragments and human-modified environments, including the peridomestic and intradomiciliary areas (19), which aligns with the scenario observed in the present study. In these environments, the abundance of organic matter resulting from subsistence agriculture and livestock farming provides a suitable habitat for the immature stages (larvae and pupae) of the species. At the same time, domestic animals, wild reservoirs, and humans serve as blood sources for the adults (20). 

Due to the biological characteristics of its vectors, the transmission of American cutaneous leishmaniasis primarily occurs in rural and wild environments; however, field activities and the place of residence are also considered risk factors (3,21). In this study, we found that *Nyssomyia intermedia* was exclusively present in the intradomiciliary area, with no captures of this species in the chicken coop. This marked anthropophilic behavior of *Nyssomyia intermedia*, including its intrusion into intradomiciliary environments, has been observed in other studies, which directly impacts the epidemiology of American cutaneous leishmaniasis (22). In the present study, it is possible that the infection occurred while the patient was sleeping, given the large number of vectors found in their bedroom. Thus, it is likely that poor socioeconomic conditions, which negatively affect housing quality, facilitate the invasion of these vectors into homes, thereby constituting a risk factor for the transmission of American cutaneous leishmaniasis (23). 

Previous studies conducted in Minas Gerais and similar regions allow some comparisons: a) the two species of Phlebotominae sand flies found in the present study are consistent with those of the biome in which the study area is inserted. *Nyssomyia intermedia* seems to be more associated with the Cerrado biome, although it may occur at a low frequency in other biomes, such as in the Atlantic Forest of Minas Gerais (24); b) studies show the domestication process of *Nyssomyia intermedia*, being often found in intradomiciliary areas (9,11). In addition, studies on blood sources have found females engorged with both human blood (*Homo sapiens*), how much chicken (*Gallus gallus*) and pigs (*Sus scrofa*), common in the peridomestic area of rural regions, which suggests the adaptation of *Nyssomyia intermedia* to the anthropized environments (27);

c) although is this is not the focus of the present study, it is likely that the epidemiological profile of American cutaneous leishmaniasis in Montezuma is similar to that observed in other regions of Brazil, exhibiting a rural occupational pattern and affecting men with low educational levels in the working-age population (28). 

Although we have not performed molecular diagnostics for the detection of Leishmania in the copies of *Nyssomyia intermedia* collected, the high frequency of this species in the household, its anthropophilic behavior, and the proven vector capacity for the transmission of *Leishmania braziliensis* highlight the crucial role of *Nyssomyia intermedia* in the transmission of American integumentary leishmaniasis.

On the other hand, *Psathyromyia pascalei* appears to have no epidemiological relevance in the transmission of leishmaniasis, and to date, it has never been found naturally infected. In addition, *Psathyromyia pascalei* maintains sylvatic habits, primarily using armadillo burrows and those of other wild animals as breeding sites (29). Unlike *Nyssomyia intermedia*, *Psathyromyia pascalei* is generally rarely sampled, occurring mainly in sylvatic environments associated with rocky formations, such as caves. However, it has also been reported in the peridomestic area (30), as noted in our study, which may indicate an adaptation of this species to human-modified environments.

Despite the limitations of this study, including the low sampling effort and the lack of diagnostic tests for Leishmania in the vectors, we emphasize the importance of this type of research, which facilitates an understanding of the ecoepidemiology of American cutaneous leishmaniasis, a neglected disease in northern Minas Gerais. Furthermore, it is important to emphasize that this research results from an entomological investigation conducted using the structure (human resources, assets, and funding) of the Brazilian Unified Health System (SUS), supported by the Regional Health Superintendent’s Office of Montes Claros, linked to the State Health Department of Minas Gerais, in collaboration with the Municipal Health Department and the Collaborating Center for Entomology. This recently implemented public policy in the state aims to optimize response capacity in the regions it serves. Therefore, it is essential to highlight the integration of all SUS entities that made this work possible.
