# Supplementary material for: Ocurrence of Nyssomyia intermedia (Diptera: Psychodidae) in intradomiciliary environments: a potential vector of american cutaneous leishmaniasis in Montezuma, 2024
Source: Epidemiol Serv Saude. 2025 Oct 27;34:e20250198. doi: 10.1590/S2237-96222025v34e20250198.en (PMC12560226; doi:10.1590/S2237-96222025v34e20250198.en)
Supplement: Translation

# Translation: Ocorrência de Nyssomyia intermedia (Diptera: Psychodidae) no intradomicílio: potencial vetor de leishmaniose tegumentar americana em Montezuma, 2024

**DOI:** 10.1590/S2237-96222025v34e20250198.pt

**Authors:** Bartolomeu Teixeira Lopes, Cirilo Henrique de Oliveira, Pedro Augusto Almeida de Souza, Rudá Mahayana Cordeiro-de-Barros, Thaynara de Jesus Teixeira, Ronaldo Medeiros dos Santos, Agna Soares da Silva Menezes, Filipe Vieira Santos de Abreu

**Affiliations:** 1 Secretaria de Saúde de Minas Gerais, Superintendência Regional de Saúde de Montes Claros, Montes Claros, MG, Brasil; 2 Instituto Federal do Norte de Minas Gerais, Laboratório de Comportamento de Insetos, Salinas, MG, Brasil; 3 Instituto Federal do Norte de Minas Gerais, Departamento de Engenharia Florestal, Salinas, MG, Brasil; 4 Secretaria Municipal de Saúde de Salinas, Centro Colaborador de Entomologia/Lacoi/IFNMG, Salinas, MG, Brasil

**Keywords:** Psychodidae, Doença Negligenciada, Entomologia, Vigilância em Saúde, Leishmaniose Tegumentar Americana

## Abstract

**Objetivo:**

Investigar a fauna de flebotomíneos na zona rural de Montezuma, Minas Gerais, após a confirmação de um caso humano de leishmaniose tegumentar americana, em uma atuação conjunta com a Superintendência Regional de Saúde de Montes Claros.

**Métodos:**

As capturas foram realizadas com armadilhas luminosas do tipo CDC (desenvolvida pelo Centers for Disease Control and Prevention), posicionadas no intradomicílio (quarto 1 e quarto 2) e no peridomicílio (galinheiro) durante duas noites consecutivas.

**Resultados:**

Ao todo, foram capturados e identificados 30 flebotomíneos, sendo 29 (96,67%) pertencentes à espécie *Nyssomyia intermedia* e 1 (3,33%) pertencente à espécie *Psathyromyia pascalei*. Surpreendentemente, todos os exemplares de *Nyssomyia intermedia* foram capturados no intradomicílio, especificamente no quarto do paciente com infecção confirmada por leishmaniose tegumentar americana.

**Conclusão:**

Devido ao grande número de espécimes capturados no interior da residência, *Nyssomyia intermedia* se destaca como um potencial vetor intradomiciliar da leishmaniose tegumentar americana. Além disso, reforçamos a importância de estudos como este, conduzidos em colaboração entre o Sistema Único de Saúde e os Centros Colaboradores de Minas Gerais, para fortalecer as ações de vigilância, promover medidas profiláticas e ampliar o conhecimento sobre a ecoepidemiologia da doença em áreas ainda pouco estudadas.

**Aspectos éticos**

Esta pesquisa utilizou bancos de dados de domínio público e anonimizados.:

## Introdução 

Os flebotomíneos (Diptera: Psychodidae, Phlebotominae) são pequenos insetos, conhecidos popularmente como mosquito-palha, que desempenham um papel importante na transmissão de patógenos para os seres humanos. Eles são responsáveis pela transmissão de diferentes espécies de protozoários intracelulares do gênero Leishmania Ross, agentes causadores das leishmanioses (1). O gênero Leishmania inclui mais de 20 espécies transmitidas entre hospedeiros mamíferos através das picadas das fêmeas de flebotomíneos durante a hematofagia. Na maioria dos casos, o ciclo de transmissão é zoonótico, envolvendo um ou mais reservatórios animais não humanos (2). No Brasil, o complexo de espécies *Lutzomyia longipalpis* atua como vetor da leishmaniose visceral, causada majoritariamente por *Leishmania* (Leishmania) infantum, enquanto *Nyssomyia intermedia* e *Nyssomyia whitmani* são os vetores primários de *Leishmania* (Viannia) braziliensis, principal espécie causadora da leishmaniose tegumentar americana (3).

A leishmaniose tegumentar americana é considerada uma doença negligenciada, potencialmente grave, caracterizada por lesões ulceradas na pele e/ou nas mucosas (4). O tratamento é disponibilizado pelo Sistema Único de Saúde (SUS), no entanto, são registradas altas taxas de abandono devido ao longo período de aplicação das injeções. A doença tem sido registrada em todos os estados brasileiros e seu padrão epidemiológico tem passado por mudanças, especialmente em relação à forma de transmissão. Observa-se um aumento no número de casos, com picos recorrentes a cada cinco anos (3). Entre 2010 e 2022, foram notificados 231.307 casos de leishmaniose tegumentar americana no Brasil, com uma média anual de 114,33 casos para cada 100 mil habitantes (5).

Minas Gerais é um dos estados brasileiros com as maiores taxas de transmissão de leishmaniose tegumentar americana. Na última década, foram registrados 16.277 casos no estado (5). Estudos anteriores confirmaram que a leishmaniose tegumentar americana é endêmica em Minas Gerais e que a macrorregião norte é uma das mais gravemente afetadas pela doença (6). Nessa região, os principais vetores são *Lutzomyia longipalpis*, associado à leishmaniose visceral (7), e *Nyssomyia intermedia*, vetor da leishmaniose tegumentar (8). Apesar da alta incidência da doença e das diretrizes estabelecidas nos manuais de controle das leishmanioses, são raros os casos em que investigações entomológicas são realizadas para identificar os vetores responsáveis pela transmissão. A escassez de profissionais capacitados e de recursos financeiros geralmente limitam a realização desse tipo de estudo, que é fundamental para a compreensão da epidemiologia dessa enfermidade.

Portanto, este trabalho descreve uma investigação entomológica realizada após a confirmação de um caso de leishmaniose tegumentar americana em um morador do município de Montezuma, no norte de Minas Gerais. O objetivo foi investigar a fauna de vetores na região afetada para identificar os flebotomíneos potencialmente envolvidos na transmissão da doença.

## Métodos 

### Área de estudo 

O estudo foi realizado em Montezuma, Minas Gerais, na comunidade rural denominada Paz Cavalo, especificamente na Fazenda Pai Pedro (15°18’00.6”S, 42°31’21.2”W). O município está localizado em uma área de transição entre os biomas Cerrado, Caatinga e Mata Atlântica, na macrorregião norte de Minas Gerais (Figura 1).

**Figura 1:** Ponto de amostragem da interseção entre três biomas brasileiros. Montezuma, 2024

Montezuma possui uma população de 6.888 habitantes e apresenta baixo índice de desenvolvimento humano municipal (IDHM 0,587) (9). O clima da região é classificado como semiárido (Aw, segundo a classificação de Köppen), com precipitação média anual de 880 mm. O regime climático é marcado por duas estações bem definidas: um verão com período chuvoso, que se estende de dezembro a março, e o predomínio de uma estação seca, prolongada nos demais meses do ano (10). A vegetação local está inserida em um ecótono entre os biomas Cerrado e Mata Atlântica, com predominância de formações vegetais e espécies características do Cerrado (11). 

A área foi selecionada para o trabalho de vigilância entomológica devido à confirmação de um caso de leishmaniose tegumentar americana em um morador da localidade, registrado em maio de 2024. Trata-se de uma região rural, com ambiente peridomiciliar utilizado para a agricultura e a pecuária de subsistência, incluindo o cultivo de hortaliças e a criação de galinhas e de porcos. O domicílio onde as coletas foram realizadas era habitado pelo paciente com diagnóstico confirmado da doença e por sua família. A residência não possuía janelas teladas nem forro no telhado e estava localizada a cerca de 20 metros de um remanescente de mata ciliar às margens do Rio Pardo.

### Coleta, processamento e identificação dos flebotomíneos

As coletas foram realizadas ao entardecer e durante a noite entre 6 e 8 de maio de 2024. O esforço amostral consistiu em três armadilhas luminosas do tipo CDC, isto é, desenvolvidas originalmente pelo Centers for Disease Control and Prevention dos Estados Unidos, sendo duas instaladas no intradomicílio (quarto 1 e quarto 2) e uma no peridomicílio, ao lado do galinheiro. As armadilhas permaneceram ativas das 18h às 7h, durante as duas noites de coleta. Os insetos capturados foram recolhidos após cada noite e armazenados em tubos, identificados por armadilha, contendo álcool a 70%. Toda a coleta foi organizada e executada por agentes treinados, pertencentes ao quadro da Superintendência Regional de Saúde de Montes Claros.

Os insetos capturados foram encaminhados ao Centro Colaborador de Entomologia LACOI/IFNMG/Salinas. Os Centros Colaboradores foram instituídos pela Secretaria de Estado de Saúde de Minas Gerais e são laboratórios vinculados a instituições do estado, com capacidade para realizar análises clínicas e científicas, prestando apoio às populações das regiões em que estão inseridos (Resolução SES/MG nº 8.441, de 9 de novembro de 2022). O LACOI/IFNMG/Salinas está credenciado na tipologia “Laboratório de Entomologia” e tem como principal atribuição a realização da taxonomia de insetos vetores, atendendo aos 86 municípios pertencentes à macrorregião de saúde do norte de Minas Gerais (CIB/SUS-MG nº 8.441, de 9 de novembro de 2022). Em laboratório, os insetos foram clarificados em solução de potassa (KOH) a 10% por 12 horas, mergulhados em ácido acético (CH₃COOH) a 100% por 5 segundos e, em seguida, em ácido acético (CH₃COOH) a 10% por 15 minutos. Posteriormente, passaram por uma série alcoólica com etanol (C₂H₅OH) nas concentrações de 70%, 90% e 100% por 10 minutos em cada etapa. Após esse processo, os insetos foram diafanizados em eugenol por 30 minutos, dissecados e montados em lâminas com bálsamo do Canadá para posterior observação ao microscópio (12). A identificação das espécies foi feita através de chave dicotômica a partir da análise dos principais caracteres morfológicos: nas fêmeas, observou-se o cibário e a espermateca; nos machos, o aparelho genital (13). Também foi utilizado o aplicativo de smartphone Lutzodex, que permite a identificação das espécies por meio de imagens reais dos exemplares (14). As abreviações dos gêneros seguiram a padronização proposta por Marcondes (15). 

Após o tratamento do paciente, a Secretaria de Estado de Saúde de Minas Gerais promoveu ações de controle vetorial com a aplicação de inseticidas e recomendou a instalação de telas nas janelas, bem como o uso de repelentes pelos moradores, especialmente durante os períodos crepusculares, conforme as orientações do Manual de Vigilância da leishmaniose tegumentar americana (3).

## Resultados 

Ao todo, foram capturados 30 flebotomíneos, sendo 29 (96,67%) pertencentes à espécie *Nyssomyia intermedia* (Figura 2, A-D) e 1 (3,33%) pertencente à espécie *Psathyromyia pascalei*. Surpreendentemente, todos os exemplares de *Nyssomyia intermedia* foram capturados no intradomicílio, sendo a maioria no quarto 1, onde foram coletados 27 indivíduos (90,00%) (Tabela 1). Destaca-se ainda a alta proporção de fêmeas capturadas no ambiente intradomiciliar, totalizando 24 indivíduos (83,00%).

**Tabela 1:** Espécies de flebotomíneos amostradas, sexo e local de coleta na Comunidade Paz Cavalo. Montezuma, 2024

Espécies	Local de coleta							
	Quarto 1		Quarto 2		Galinheiro			
	Fêmea	Macho	Fêmea	Macho	Fêmea	Macho	Total	%
*Nyssomyia intermedia* (Lutz & Neiva, 1912)	22	5	2	0	0	0	29	96,67
*Psathyromyia pascalei* (Coutinho & Barreto, 1940)	0	0	0	0	1	0	1	3,33
Total	22	5	2	0	1	0	30	100,0

**Figura 2:** Detalhes anatômicos das estruturas reprodutivas de *Nyssomyia intermedia*: (A) abdômen da fêmea visualizado em posição ventral (aumento de 200 vezes); (B) espermateca da fêmea com anatomia compatível com *Nyssomyia intermedia* (aumento de 400 vezes); (C) região terminal do abdômen do macho (aumento de 200 vezes); (D) extremidade do filamento genital do macho com anatomia compatível com *Nyssomyia intermedia* (aumento de 400 vezes). Brasil, 2024

O material foi recebido pelo Centro Colaborador em 16 de maio de 2024, e os laudos taxonômicos foram emitidos em 18 e 19 de maio de 2024 (até 72 horas após o recebimento). Isso resultou em uma resposta de controle vetorial na área afetada, incluindo atividades de educação ambiental e sanitária, coordenadas pelos agentes de saúde e de combate às endemias em Montezuma. Desde então, nenhum outro caso humano foi registrado na região.

## Discussão 

A distribuição geográfica das leishmanioses no Brasil revela que sua epidemiologia é influenciada por aspectos ambientais, climáticos e socioeconômicos (16). A leishmaniose tegumentar americana, doença típica de ambientes rurais, tem sofrido mudanças em sua epidemiologia, impulsionada por modificações socioambientais, como o desmatamento e o processo migratório de populações humanas provenientes de áreas rurais endêmicas para a doença (3). O surgimento desses novos fatores de risco tem favorecido o contato humano-vetor e gerado novos cenários epidemiológicos, aumentando a incidência dessas doenças (1). 

*Nyssomyia intermedia* apresenta ampla distribuição geográfica no Brasil, ocorrendo desde o Piauí até o Rio Grande do Sul, e em partes da Argentina e da Bolívia, sendo predominantemente encontrada em regiões quentes e úmidas (17). Além disso, essa espécie já foi encontrada naturalmente infectada com *Leishmania braziliensis*, sendo considerada um dos principais vetores da leishmaniose tegumentar americana (8). Adicionalmente, *Nyssomyia intermedia* apresenta comportamento altamente antropofílico, sendo geralmente abundante no peridomicílio. Ela foi a espécie predominante em surtos de leishmaniose tegumentar americana em cidades de Minas Gerais, como Araçuaí, Lassance e Curvelo (18). 

É importante ressaltar que a paisagem da área de estudo, caracterizada pela proximidade com remanescentes de vegetação natural, favorece tanto a ocorrência de *Nyssomyia intermedia* quanto o seu contato com reservatórios silvestres de Leishmania, como gambás e roedores, além de outros hospedeiros domésticos, como cães e seres humanos (3). Nas últimas décadas, tem sido demonstrado que *Nyssomyia intermedia* tende a ocorrer em altas densidades em fragmentos de floresta secundária e em ambientes modificados pelo homem, incluindo o perímetro e o interior das residências (19), o que coincide com o cenário deste estudo. Nesses ambientes, a abundância de matéria orgânica, proveniente da agricultura e da pecuária de subsistência, serve de habitat para os imaturos (larvas e pupas) da espécie, enquanto animais domésticos, reservatórios silvestres e seres humanos fornecem alimento para os adultos (20). 

Devido às características biológicas de seus vetores, a transmissão da leishmaniose tegumentar americana ocorre principalmente em ambientes rurais e silvestres, no entanto, atividades em campo são consideradas fatores de risco, assim como o local de residência (3,21). Em nossa amostragem, registramos a ocorrência de *Nyssomyia intermedia* exclusivamente no intradomicílio, sem capturas dessa espécie no galinheiro. Esse comportamento antropofílico marcante de *Nyssomyia intermedia*, incluindo a invasão intradomiciliar, foi observado em outros estudos, o que impacta diretamente a epidemiologia da leishmaniose tegumentar americana (22). Neste estudo, é possível que a infecção tenha ocorrido enquanto o paciente dormia, dado o grande número de vetores encontrados em seu quarto. Dessa forma, é provável que as condições socioeconômicas precárias, que impactam negativamente a qualidade das habitações, favoreçam a invasão desses vetores nos domicílios, constituindo, assim, um fator de risco para a transmissão da leishmaniose tegumentar americana (23). 

Estudos anteriores realizados em Minas Gerais e em regiões semelhantes permitem algumas comparações: (i) As duas espécies de flebotomíneos encontradas neste presente estudo condizem com a do bioma em que a área de estudo está inserida. *Nyssomyia intermedia* parece estar mais associada ao bioma Cerrado, embora possa ocorrer em baixa frequência em outros biomas, como na Mata Atlântica de Minas Gerais (24); (ii) Os estudos evidenciam o processo de domesticação de *Nyssomyia intermedia*, sendo frequentemente encontrada no intradomicílio (9,11). Além disso, estudos sobre fontes sanguíneas encontraram fêmeas ingurgitadas tanto com sangue humano (*Homo sapiens*), quanto de galinha (*Gallus gallus*) e de porcos (*Sus scrofa*), comuns no peridomicílio das zonas rurais, o que sugere a adaptação de *Nyssomyia intermedia* a ambientes antropizados (27); (iii) Embora não seja o foco deste estudo, é provável que o perfil epidemiológico da leishmaniose tegumentar americana em Montezuma seja semelhante ao observado em outras regiões do Brasil, exibindo um caráter ocupacional rural e acometendo homens com baixa escolaridade na idade produtiva (28). 

Embora não tenhamos realizado diagnósticos moleculares para a detecção de Leishmania nos exemplares de *Nyssomyia intermedia* coletados, a alta frequência dessa espécie no intradomicílio, o seu comportamento antropofílico e a capacidade vetorial comprovada para a transmissão de *Leishmania braziliensis* evidenciam o papel crucial de *Nyssomyia intermedia* na transmissão da leishmaniose tegumentar americana.

Por outro lado, *Psathyromyia pascalei* parece não ter relevância epidemiológica na transmissão das leishmanioses e, até o momento, nunca foi encontrada naturalmente infectada. Além disso, *Psathyromyia pascalei* mantém hábitos majoritariamente silvestres, utilizando principalmente buracos de tatu e de outros animais selvagens como criadouros (29). Ao contrário de *Nyssomyia intermedia*, *Psathyromyia pascalei* é geralmente pouco amostrada, ocorrendo principalmente em ambientes silvestres associados a formações rochosas, como cavernas. No entanto, também já foi registrada no peridomicílio (30), como observado em nosso estudo, o que pode indicar uma adaptação dessa espécie ao ambiente modificado pelo homem.

Apesar das limitações deste trabalho, como o baixo esforço amostral e a ausência de exames diagnósticos para Leishmania nos vetores, destacamos a importância desse tipo de estudo, que facilita a compreensão da ecoepidemiologia da leishmaniose tegumentar americana, uma doença negligenciada, no norte de Minas Gerais. Além disso, é relevante ressaltar que esta pesquisa é o resultado de uma investigação entomológica realizada com a estrutura (recursos humanos, patrimônio e custeio) do SUS, com apoio da Superintendência Regional de Saúde de Montes Claros, vinculada à Secretaria Estadual de Saúde de Minas Gerais, em colaboração com a Secretaria de Saúde Municipal e do Centro Colaborador de Entomologia. Essa política pública, recém-implantada no estado, tem como objetivo otimizar a capacidade de resposta nas regiões atendidas. Portanto, é fundamental destacar a integração de todos os entes do SUS, que viabilizaram a realização deste trabalho.
